# Targeted Metabolic Profiles of the Leaves and Xylem Sap of Two Sugarcane Genotypes Infected with the Vascular Bacterial Pathogen *Leifsonia xyli* subsp. *xyli*

**DOI:** 10.3390/metabo11040234

**Published:** 2021-04-12

**Authors:** Fernanda R. Castro-Moretti, Jean-Christophe Cocuron, Mariana C. Cia, Thais R. Cataldi, Carlos A. Labate, Ana Paula Alonso, Luis E. A. Camargo

**Affiliations:** 1 Department of Biological Sciences, University of North Texas, 1504 W Mulberry St., Denton, TX 76201, USA; Fernanda.Moretti@unt.edu (F.R.C.-M.); Jeanchristophe.Cocuron@unt.edu (J.-C.C.); Anapaula.Alonso@unt.edu (A.P.A.); 2 BioDiscovery Institute, University of North Texas, 1504 W Mulberry St., Denton, TX 76201, USA; 3 Centro de Tecnologia Canavieira, Fazenda Santo Antonio, Piracicaba 13418-970, Brazil; cicarellicia@gmail.com; 4 Department of Genetics, Luiz de Queiroz College of Agriculture, University of São Paulo, Avenue Pádua Dias 11, Piracicaba 13418-900, Brazil; thais.cataldi@usp.br (T.R.C.); calabate@usp.br (C.A.L.); 5 Department of Plant Pathology and Nematology, Luiz de Queiroz College of Agriculture, University of São Paulo, Avenue Pádua Dias 11, Piracicaba 13418-900, Brazil

**Keywords:** bacterial pathogen, metabolomics, plant defense, ratoon stunting disease, *Saccharum* spp.

## Abstract

Ratoon stunt (RS) is a worldwide disease that reduces biomass up to 80% and is caused by the xylem-dwelling bacterium *Leifsonia xyli* subsp. *xyli*. This study identified discriminant metabolites between a resistant (R) and a susceptible (S) sugarcane variety at the early stages of pathogen colonization (30 and 120 days after inoculation—DAI) by untargeted and targeted metabolomics of leaves and xylem sap using gas chromatography-mass spectrometry (GC-MS) and liquid chromatography-tandem mass spectrometry (LC-MS/MS), respectively. Bacterial titers were quantified in sugarcane extracts at 180 DAI through real-time polymerase chain reaction. Bacterial titers were at least four times higher on the S variety than in the R one. Global profiling detected 514 features in the leaves and 68 in the sap, while 119 metabolites were quantified in the leaves and 28 in the sap by targeted metabolomics. Comparisons between mock-inoculated treatments indicated a greater abundance of amino acids in the leaves of the S variety and of phenolics, flavonoids, and salicylic acid in the R one. In the xylem sap, fewer differences were detected among phenolics and flavonoids, but also included higher abundances of the signaling molecule sorbitol and glycerol in R. Metabolic changes in the leaves following pathogen inoculation were detected earlier in R than in S and were mostly related to amino acids in R and to phosphorylated compounds in S. Differentially represented metabolites in the xylem sap included abscisic acid. The data represent a valuable resource of potential biomarkers for metabolite-assisted selection of resistant varieties to RS.

## 1. Introduction

Ratoon stunt (RS) occurs worldwide as a serious threat in all sugarcane-producing countries [1]. This disease was firstly described in Australia in 1944, but its etiology was only established in 1980 [2,3]. Reported annual losses to RS range from 1 to 11 million US dollars [4,5,6] and its prevalence in commercial fields has been recently reported to be higher than 60% in Brazil and China [7,8]. However, as the methods used to detect its causal agent, the gram-positive and fastidious bacterium *Leifsonia xyli* subsp. *xyli* (Lxx), in commercial fields are prone to errors, it is highly probable that losses and prevalence estimates are underestimated [9]. The major symptoms of RS are shorter internodes and thinner stalks that develop most visibly in ratoon plants, i.e., plants that grow from the stubble after harvesting, leading to a marked reduction in biomass production over successive cropping [10]. Diagnosis of RS based on symptoms alone is difficult, as they can be easily mistaken for symptoms caused by environmental factors that affect plant growth.

*Leifsonia xyli* subsp. *xyli* colonizes the xylem vessels and the bundle sheath cells that surround the leaf vascular system of sugarcane [11,12,13] and is regarded as an obligatory endophytic organism that grows to parasitic levels depending on biotic and abiotic factors that are yet poorly understood [14]. The most-used method for controlling RS is to heat-treat stalk cuttings used as propagative material with hot water, but the efficacy of this method in killing the bacteria is variable and it may also affect the germinability of the buds [15,16]. Thus, because Lxx is mechanically transmitted to healthy plants through harvesting blades and tools contaminated with juices of diseased plants, RS is very difficult to control once it is established in commercial fields, as the number of infected plants tends to increase along consecutive harvests [17].

Genetic control of RS by selecting genotypes that restrict the multiplication of the bacterium is a promising strategy since sugarcane genotypes vary in relation to the levels of Lxx colonization of their tissues, and higher bacterial densities are associated with higher losses [18,19,20]. However, phenotypic selection of resistant genotypes based on inoculation trials is not feasible because symptoms take too long to manifest. An alternative would be to quantify Lxx *in planta*, but this requires serological or molecular methods that add significant costs to the breeding programs. For instance, it is required to screen approximately 250 thousand seedlings in order to develop a single commercial variety just by selecting for higher sugar content alone and not considering other traits [21].

In grasses, the culm is sectioned by the nodes, where some xylem vessels have their diameter reduced when they branch out to the leaves, thus restricting water flow, whereas some pass through the nodes without branching, and therefore, have larger diameters. It has been demonstrated that the water flow though the nodes differs among sugarcane varieties, being higher in susceptible genotypes, suggesting that these have higher frequencies of large continuous vessels that facilitate the systemic colonization of the plant by the bacteria [22,23]. However, in addition to this structural resistance, plant defenses can also be of biochemical nature, mostly conferred by secondary antimicrobial metabolites [22]. These could be constitutively present in the host tissues, thereby forming natural barriers to prevent or reduce pathogenesis [23] or induced after infection, i.e., their production and accumulation are enhanced when the host recognizes the pathogen through signaling mechanisms [24]. Constitutive or induced biochemical mechanisms involved in resistance or susceptibility of sugarcane to Lxx have not yet been studied and the possibility of identifying plant metabolites that control bacterial growth as a mechanism of resistance is appealing, since they can be used as biomarkers to select resistant genotypes in breeding programs. Indeed, an unknown xylem sap metabolite of maize, possibly a non-reducing sugar, was found to enhance the growth of the Lxx-closely related organism *Leifsonia xyli* subsp. *cynodontis*, an endophyte of grasses, including sugarcane [25].

The main objective of this study was to compare the metabolic profiles of a resistant and a susceptible sugarcane variety in response to variations in bacterial titers within their tissues over time, following a similar experimental design of a previous transcriptomic and proteomic study on the same pathosystem [14]. Plants with low bacterial levels were mock-inoculated or inoculated with Lxx to simulate a bacterial increase from endophytic to parasitic levels. Leaves and xylem sap samples were collected 30 and 120 days after inoculation (DAI) and subjected to untargeted and targeted metabolic profiling. Distinct profiles were detected between varieties, sample origins (leaves and sap), and inoculation treatments.

## 2. Results

### 2.1. Lxx Titer Is Higher in Inoculated S Plants

**Figure 1:**
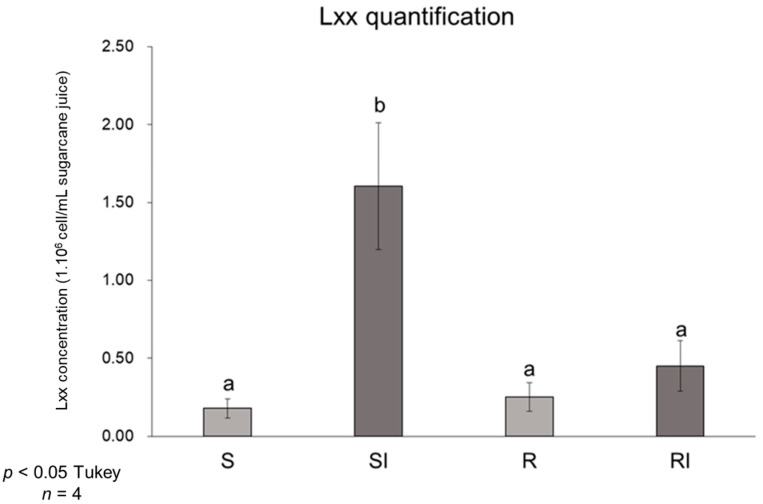
Lxx concentration (×10^6^ bacterial cells/mL of sugarcane extract) at 180 days after inoculation (DAI) in mock-inoculated or Lxx-inoculated plants of the susceptible (S and SI, respectively) and resistant (R and RI, respectively) varieties determined by qPCR. Letters indicate significantly different means according to Tukey’s test (*p* < 0.05). Bars represent standard errors (*n* = 4).

The S and R varieties were inoculated with Lxx or mock-inoculated with a NaCl solution (0.85%). For metabolomic profiling, samples were collected at 30 and 120 DAI. The concentration of the inoculum was 6.5 × 10^10^ CFU mL^−1^ as estimated by plate counting of the serial dilution (data not shown). Quantitative PCR of the vascular fluid collected from the stems at 180 DAI using primers specific for Lxx showed four-fold higher bacterial titers (*p* < 0.05) in the inoculated plants of the S variety compared to the mock-inoculated control ones, whereas for the R variety, there was no significant increase in titers in the Lxx-inoculated plants compared to the mock-inoculated ones (Figure 1).

### 2.2. Untargeted Metabolomics Detected Sugars, Organic Acids, and Phenolic Compounds in Sugarcane Leaves and Sap

Untargeted metabolomics was performed in leaves and xylem sap samples collected at 120 DAI from the S and R varieties inoculated or not with Lxx to compare their global metabolic profiles. A total of 514 and 68 features were detected across treatments in the leaves and in the sap, respectively. Using the NIST library, it was possible to identify sugars, such as trehalose and sucrose; organic acids, such as malic, quinic, stearic and palmitic acids, and inositol; and phenolic compounds, such as chlorogenic (isomers of caffeoylquinic acid) and coumaric acids (Table S1). Sucrose, for instance, was relatively more abundant in SI, R, and RI leaves. Interestingly, phenolic compounds, which are metabolites related to plant defense, were more abundant in R leaves. Nonetheless, most of the features could not be identified either because there was no match with NIST or the probability of the match was lower than 70% (data not shown). As this analysis revealed quantitative rather than qualitative differences among treatments, a targeted approach was used to determine the absolute levels of amino acids, sugars, sugar alcohols, organic acids, phosphorylated compounds, phenolics, flavonoids, and hormones in the leaves. In the sap, only sugars and phenolics were targeted since the detection levels of other metabolites were low (Table S1).

### 2.3. Targeted Metabolomics of Leaves and Xylem Sap

**Figure 2:**
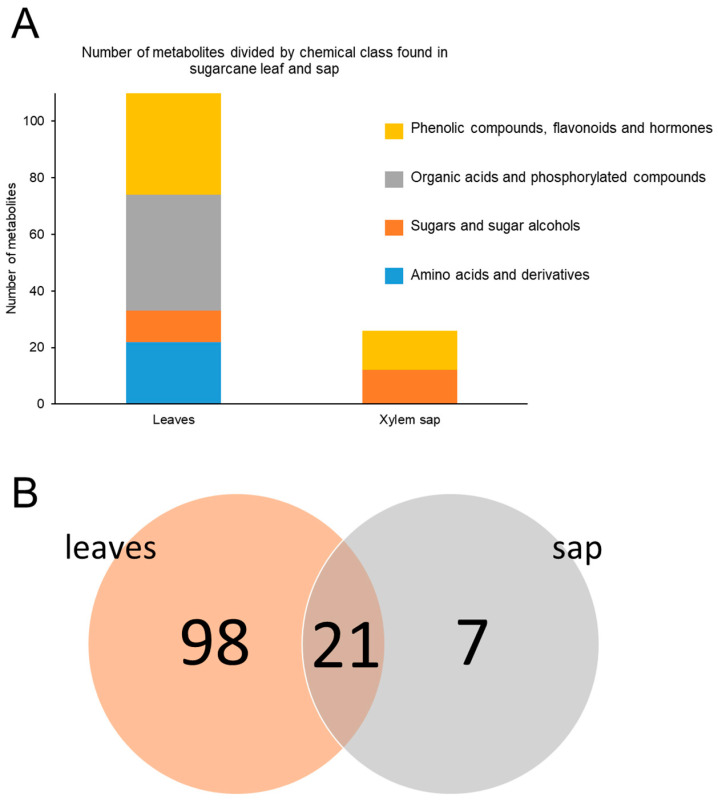
Number of compounds detected and quantified by targeted metabolomics grouped according to their chemical class (**A**) and numbers of unique or shared compounds in the leaves and xylem sap (**B**).

Targeted LC-MS/MS analyses were performed on samples from sugarcane plants mock-inoculated or inoculated with Lxx, collected at 30 and 120 DAI. In total, 119 metabolites were quantified in the leaves: 23 amino acids and their derivatives, 13 sugars and sugar alcohols, 49 organic acids and phosphorylated compounds, and 34 phenolic compounds, flavonoids, and hormones (Figure 2A). In the xylem sap, 28 metabolites were quantified: 12 sugars and sugar alcohols and 16 phenolic compounds, flavonoids, and hormones (Figure 2A). Eighty-nine metabolites were unique to leaf extracts, while only five were unique to the xylem sap (Figure 2B).

### 2.4. Small Variations in the Composition of the Xylem Sap in Relation to Plant Genotype, Time, and Bacterial Inoculation

All metabolites detected in the xylem sap were in low concentrations compared to leaf extracts (Table S2). However, most metabolites were more abundant at 120 DAI than at 30 DAI regardless of the plant genotypes and inoculation treatment (Table S2). Glycerol, sorbitol, sinapaldehyde, and luteolin-7-O-glucoside showed differential accumulation (*p* < 0.05) between S and R mock-inoculated plants. Few metabolic differences were detected between mock and Lxx-inoculated plants within varieties. In the S one, the concentration of abscisic acid (ABA) was lower at 30 DAI, while ferulic acid was higher at 120 DAI in Lxx-inoculated plants with higher bacterial titers compared to the mock-inoculated ones. In the R variety, sinapaldehyde was less and 4-hydroxybenzoic acid was more abundant (*p* < 0.05) in Lxx-inoculated plants at 30 DAI (Table S2).

### 2.5. Resistant and Susceptible Mock-Inoculated Plants Have Distinct Leaf Metabolic Profiles and Change with Time

**Figure 3:**
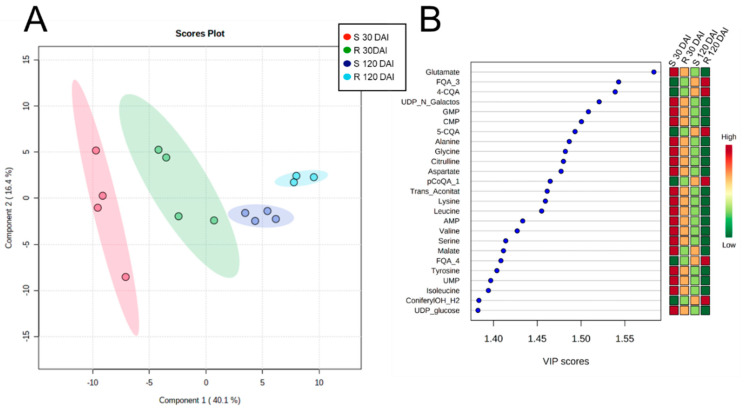
PLS-DA cluster (**A**) and the 20 most important features (**B**) of leaves extracts from mock-inoculated S and R varieties collected at 30 and 120 DAI quantified by targeted metabolomics. Shaded red, green, blue, and cyan regions in (**A**) represent 95% confidence intervals. Colors in the Variable Importance in Projection (VIP) plot represent relative abundance. Abbreviations: FQA_3, feruloylquinic acid isomer 3; 4-CQA, 4-caffeoylquinic acid; UDP_N_Galactos, uridine diphosphate-N-galactosamine; GMP, guanosine monophosphate; CMP, cytidine monophosphate; 5-CQA, 5-caffeoylquinic acid; pCoQA_1, isomer 1 of p-coumaroylquinic acid; Trans_Aconitat, trans-aconitate; AMP, adenosine monophosphate; FQA_4, isomer 4 of feruloylquinic acid; UMP, uredine monophosphate, ConiferylOH_H2, coniferyl alcohol (-H_2_O); UDP_glucose, uracil diphosphate glucose.

**Figure 4:**
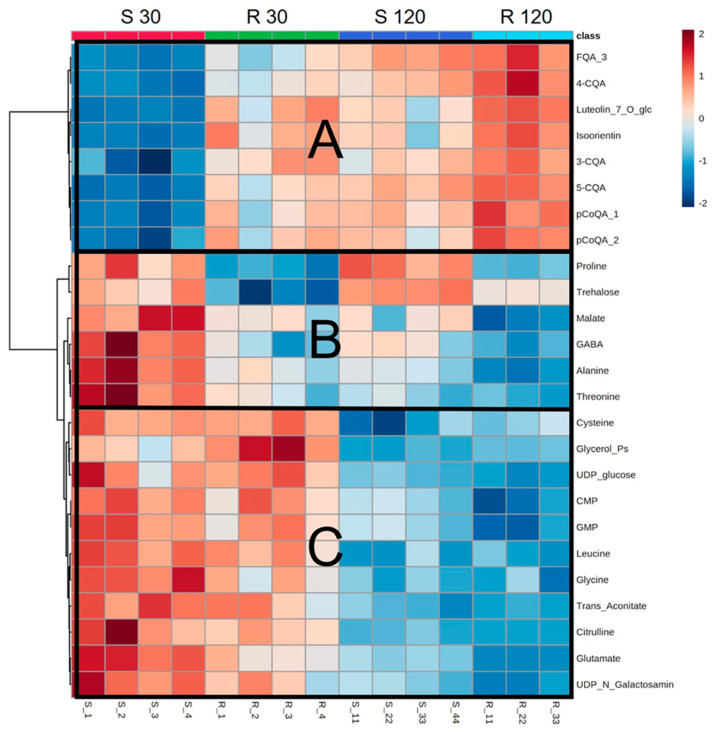
Heatmap showing the top 25 ANOVA compounds of leaf extracts quantified by targeted metabolomics from the mock-inoculated plants of the S and R varieties collected at 30 and 120 DAI. Colors represent metabolite relative intensity, where red represents high values and blue represents low values, and fold changes are represented by the color scale. Highlighted sections represent groups of compounds that are low in S30 and high in S120 (**A**), high in S 30 (**B**), and higher at 30 vs. 120 DAI (**C**). Abbreviations: FQA_3, feruloylquinic acid isomer 3; 4-CQA, 4-caffeoylquinic acid; Luteolin_7_O_glc, luteolin-7-O-glucoside; 3-CQA, 3-caffeoylquinic acid; 5-CQA, 5-caffeoylquinic acid; pCoQA_1, p-coumaroylquinic acid isomer 1; pCoQA_2, p-coumaroylquinic acid isomer 1; GABA, gamma aminobutyric acid; Glycerol_Ps, glycerol phosphates; UDP_glucose, uracil diphosphate glucose; CMP, cytidine monophosphate; GMP, guanosine monophosphate; UDP_N_Galactosamin, uridine diphosphate-N-galactosamine.

Partial least squares discriminant analysis (PLS-DA) of the metabolomic profiles of mock-inoculated plants of both varieties revealed four distinct clusters, clearly indicating metabolic differences due to time and plant genotype (Figure 3), which can be seen in the heatmap analyses too (Figure 4, and Table S3). In both genotypes, the levels of phenolics and flavonoids increased over time (Figure 4A), whereas the amino acids, phosphorylated compounds, and organic acids decreased (Figure 4B,C).

Contrasting metabolite abundances between mock-inoculated S and R plants (S × R contrasts; Tables S3 and S4) unveiled striking differences, especially in metabolite classes with known role in biotic interactions: on average, S presented 45% more amino acids than R, ranging from 15% (histidine at 120 DAI) to 66% (alanine at 30 DAI), with the only exception being glutamine, which was less abundant at 120 DAI. By contrast, except for caffeic acid, R accumulated on average 60% more phenolics, ranging from approximately 14% (5-chlorogenic acid at 120 DAI) to 91% (4-hydroxybenzoic acid at 30 DAI), 62% more flavones (from 39% of isoorientin to 88% of luteolin-7-o-glucoside), and 81% more of the hormone salicylic acid—SA (30 DAI). Moreover, in most comparisons, R presented lower accumulation of organic acids and phosphorylated compounds.

### 2.6. Sugarcane Varieties Respond Differently to Pathogen Inoculation

**Figure 5:**
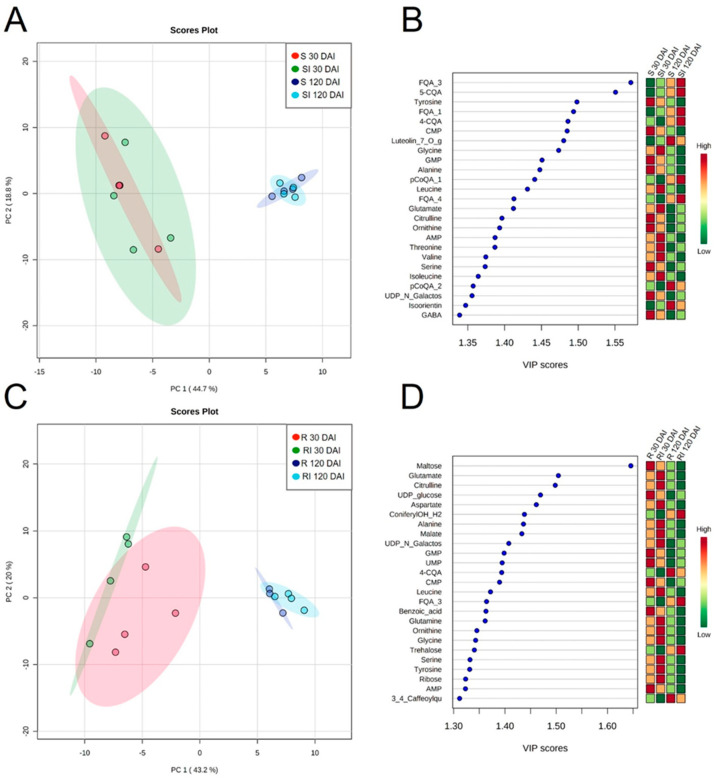
PCA clusters (**A**,**C**) and PLS-DA of the 25 most important features (**B**,**D**) of susceptible (S) and resistant (R) genotypes mock-inoculated or Lxx-inoculated (I) collected at 30 and 120 DAI quantified by targeted metabolomics. Biological replicates are represented by dots and shaded red, green, blue, and cyan ellipses represent 95% confidence intervals. Colors in the Variable Importance in Projection (VIP) plot represent relative intensity, where red represents the high values and green represents the low values. Abbreviations: FQA_3, feruloylquinic acid isomer 3; 5-CQA, 5-caffeoylquinic acid; CMP, cytidine monophosphate; Luteolin_7_O_glc, luteolin-7-O-glucoside; GMP; pCoQA_1; FQA_4; AMP; pCoQA_2; UDP_N_Galactos; GABA; UDP_glucose, ConyferylOH_H2; UMP; 4-CQA; FQA_3; AMP; 3_4_Caffeoylqu, 3,4-caffeoylquinic acid.

The second level of contrasts focused on the responses of each variety to inoculation with the pathogen by comparing mock and Lxx-inoculated plants (S × SI and R × RI). Principal component analyses (PCAs) did not separate the S × SI nor R × RI treatments into distinct clusters (Figure 5A,C), indicating that most changes were related to time after inoculation. However, 15 and 19 differentially accumulated compounds were identified in response to inoculation in S and R, respectively (Tables S4–S6). Moreover, the responses varied in metabolite composition and timing. In S, most changes occurred at 120 DAI for organic acids and phosphorylated compounds, whereas in R, changes were detected at both times, predominantly among the amino acids and sugars. At 30 DAI, only tartaric acid and luteolin-7-O-glucoside were significantly higher (*p* > 0.05) in S Lxx-inoculated plants (Figure 5B, Tables S4 and S5). However, at 120 DAI, cysteine, lysine, phosphorylated compounds (CTP, UDP, UTP, ATP, CDP), trans and cis-aconitate, malonic acid, apigenin-7-O-glucoside, and feruloylquinic acid isomer 2 (FQA-2) were significantly greater in Lxx-inoculated leaves; proline was the only metabolite whose levels went down (Tables S4 and S5).

There was less overlap between the PCA clusters in the R × RI contrast in comparison to S × SI (Figure 5A,C). Interestingly, bacterial inoculation increased the abundance of most of the compounds at 30 DAI, but decreased at 120 DAI (Figure 5D, Tables S4 and S6). The foremost among these differences was found in the amino acid class whose abundances increased at 30 DAI (alanine, valine, homoserine, threonine, and leucine) but decreased at 120 DAI (cysteine, glutamate, citrulline, homoserine, and threonine). The exceptions were cysteine and lysine; the accumulation pattern of cysteine differed from the rest of the amino acids as it decreased in the earlier time and later increased together with lysine. Other differentially accumulated metabolites were the more abundant inositol, malate, and malonic acid at 30 DAI and the less abundant maltose, IMP, caffeic acid (30 DAI), arabitol, ribose, and ADP (120 DAI; Table S4).

## 3. Discussion

### 3.1. The Parasitic Behavior of Leifsonia xyli subsp. xyli Is a Challenge for Omics Studies

The accumulation of deleterious mutations in essential genes predicted from the analysis of its genome sequence suggestive of a gene decay process and the fact that Lxx lives only within sugarcane indicate that it is an obligate endophyte of *Saccharum* spp. [13,26]. However, even though it colonizes sugarcane asymptomatically, it can cause serious yield losses if its population grows to parasitic levels. This dual endophytic/parasitic lifestyle is not uncommon among vascular pathogens [14], and understanding the factors that stimulate Lxx growth in sugarcane is key to devising methods for RS control. Plant genotype [27], water stress [28,29], and successive cropping [10] are contributing variables to RS severity, but the exact mechanisms involved are yet to be clarified. To date, however, there have been no reports on host determinants of susceptibility/resistance. Given the dual relationship of Lxx with sugarcane, and according to our previous transcriptomic study [14], in the S × SI and R × RI comparisons, we contrasted plants with different levels of infection rather than uninfected/infected to understand the alterations caused by changes in bacterial titers. In addition, by comparing mock-inoculated plants of the two varieties (S × R comparison), we identified a set of candidate metabolites present at the lowest level of infection that may stimulate or inhibit Lxx population increase. The resistance levels of the varieties were confirmed by quantifying bacterial titers via real-time qPCR. As the xylem vessels are the main niche of Lxx, quantification was done in the vascular extracts only at the end of the experiment (180 DAI, i.e., 60 days after the last sampling) because it is a destructive sampling. Therefore, due to this experimental limitation, the reported metabolic changes cannot be correlated to bacterial titers determined at the times of sample collection, but rather, it is assumed that they reflect metabolic adaptations over a colonization period that either restrict or allow pathogen growth depending on the variety. In fact, bacterial populations in the susceptible genotype (CB49-260) were four times greater than in the resistant one and the mock-inoculated plants of both varieties, confirming its susceptibility. By contrast, plants of the more resistant genotype (SP80-3280) were able to keep the initial levels of Lxx under control, as there were no significant differences between the mock and Lxx-inoculated treatments. Thus, differently than CB49-260, SP80-3280 has the capacity to check pathogen growth.

### 3.2. Leaves Had a Greater Number of Identified Features Than the Xylem Sap

Global profiling was performed at 120 DAI and revealed the presence of amino acids, sugars, organic acids, and phenolics in the sap or leaves. The number of metabolic studies in sugarcane is limited, especially in the field of pathogen interaction. Nonetheless, the compound classes detected in this analysis were the same as those reported in previous studies on sugarcane and other grasses using different detection methods [30,31]. The susceptible variety had higher abundances of organic acids, whereas the resistant plants had greater levels of sugars in the leaves. To date, no studies on sugarcane xylem sap collected from guttation drops have been performed. Although the results indicated fewer metabolites compared to the leaves, it was still possible to detect higher fructose levels in the susceptible genotype as opposed to what was found in the leaves of plants inoculated with Lxx. Detecting metabolites in the xylem sap is challenging due to their low concentrations—most molecules were found to be close to the limit of detection—and the great variability among replicates, even though the samples were collected in the same day and time. Thus, the detected compounds might not represent the whole set of metabolites. Our methodology involved collecting drops from the hydathodes, which required repetitive sampling because of the small volume obtained in each collection. As the guttation rate varies among plants, it is possible that the collected drops had different concentrations of metabolites, thus leading to variation between plants [32]. Furthermore, most metabolites found in the sap were unknown to the NIST library, meaning that there are still potential biochemical compounds that need to be identified. Similarly, another study that compared the global profiles of xylem saps from different plant species subjected to iron deficiency related that more than 70% of the detected compounds were unknown [33]. As the untargeted metabolomic profiling of leaves and sap identified sugars, organic acids, and phenolics, these classes of metabolites were targeted by LC MS/MS and quantified from samples collected at 30 and 120 DAI.

### 3.3. The Susceptible Variety Had Higher Accumulation of Bacterial Growth Enhancers and Fewer Inhibitors

Comparisons of S and R by targeted analysis of leaf tissue unveiled differential accumulation of compounds that satisfactorily explain their contrasting levels of resistance to pathogen growth. The susceptible genotype showed greater content of many amino acids, which is a relevant finding since besides being key elements for protein production, amino acids are essential for regulating many bacterial processes [34], such as quorum sensing [35] and cell wall integrity [36]. Similarly, the sugars arabinose/xylose and trehalose were also more abundant in the susceptible genotype, approximately two to 10 times higher, respectively. Interestingly, in silico analysis of the genome sequence of Lxx indicated that it has several ABC transporters, including for xylose, arabinose, and trehalose [11]. Xylose and trehalose are of special interest as Lxx has: (i) the *xylA* (xylose isomerase) and *xylB* (xylulose-5-kinase) genes of the xylose isomerase pathway [37] for the assimilation of xylose by the pentose phosphate pathway and (ii) *malE*, encoding a maltose/trehalose-binding protein for the recruitment of this osmoprotectant from sugarcane under drought conditions for rehydration [38]. By contrast, the resistant variety had a higher accumulation of chlorogenic acids—CGA, 3, 4, and 5 caffeoylquinic acids (3, 4 and 5-CQA), feruloylquinic acid isomer 3 (FQA-3), isomers 1 and 2 of p-coumaroylquinic acid (pCoQA1 and 2), and flavonoids (isoorientin, rhamnosylisoorientin, eriodictyol, and luteolin-7-O-glucoside), which are known for their antimicrobial activity and for their role in plant defense. It has been suggested that the antibacterial activity of chlorogenic acids results from alterations in the cell membrane permeability, leading to irreversible leakage of the cytoplasm [39]. In potato, higher concentrations of CGA and total soluble phenols correlated with higher levels of resistance to bacterial species that cause soft rot of tubers [40]; genetically-engineered tomato plants producing higher levels of 3 and 5 CQA presented reduced levels of infection by *Pseudomonas syringae* [41]. Flavonoids are widely known as antioxidative protectants in plants, besides playing a direct role against biotic stressing agents [42]. Additionally, the data revealed that salicylic acid, a central hormone of plant immunity and protection from oxidative stress caused by abiotic and biotic agents [43,44], was more abundant in resistant leaf samples.

In the xylem sap, the R variety presented more sinapaldehyde, an intermediate in the synthesis of lignin, and of the flavonoid luteolin-7-O-glucoside, which was also more abundant in the leaves. Moreover, it also showed a higher abundance of glycerol and sorbitol. A direct role of these compounds in the context of the interaction of sugarcane with Lxx is less clear due to the lack of information of their physiological roles. Sorbitol, an end product of photosynthesis, is transported through the phloem to sink tissues, where it is converted to fructose, but its presence in the xylem indicates that it is reallocated from the roots to the leaves, where it may provide additional carbon for growth. Nonetheless, differences in the abundance of fructose in the leaves would be expected between S and R, which was not the case. In view of recent findings, an alternative explanation is that both sorbitol and glycerol mediate the activation of the immune system as exogenous applications of these compounds increase resistance against fungi and bacteria [45,46,47,48]. Moreover, sorbitol modulates the expression of genes coding for leucine-rich repeat (NLR) and glycine rich proline domains found in proteins of the immune system of plants and animals [45]. It has been shown that exogenous pre-treatment of plants with these compounds increases resistance against fungi and bacteria [45,46,47,48]. Given the established roles of amino acids, phenolics, and flavonoids in plant-pathogen interactions, we hypothesize that the differential abundances of these metabolites in the leaves and sap of the two genotypes are key determinants of bacterial growth, and as such, they represent an interesting source of metabolic markers to screen for resistant sugarcane varieties.

### 3.4. Sugarcane Varieties Show Distinct Metabolic Responses to Bacterial Inoculation in the Leaves

By comparing mock- and pathogen-inoculated plants, a clear differential response of the varieties became evident. In the S genotype, where bacterial population increased over time after inoculation, most of the changes in metabolite abundances (13 of the 15, or 87%) occurred at 120 DAI, whereas in R, most of the changes (14 of 22, or 64%) were detected at 30 DAI, indicating a different timing of metabolic responses to pathogen inoculation. In S, changes predominated in phosphorylated compounds, which were detected at a higher abundance in Lxx-inoculated plants, indicating either increased synthesis rates or reduced transportation of these metabolites to sink organs in response to increased bacterial titers. However, the latter scenario seems more likely to happen because of the known negative effects of Lxx on photosynthesis [49].

In the R genotype, most changes were observed in the amino acid class, whose levels followed an interesting pattern: they increased at the earlier time but decreased later. The data agree with a previous work that reported increased AA content at later times (150, 180, and 210 DAI) after inoculation with Lxx [49]. Recently, upregulation of primary metabolism in response to pathogen infection has been given more attention, as there is mounting evidence of the involvement of these pathways, including those of amino acids, lipids, and carbohydrates as modulators of plant defenses [50]. From the pathogen perspective and considering the multiple roles of amino acids in bacterial pathogenicity, it would be expected that this momentary increase in AA would stimulate bacterial growth. However, population levels do not increase, perhaps because the induction does not rise their levels to the optimal necessary for growth as in the S genotype and more importantly because the availability of these metabolites diminish later after a metabolic reprograming exclusive of the R genotype, which further hinders the colonization process. Cysteine is an intriguing exception, as it presented an opposite accumulation pattern, i.e., its levels decreased by 32% at first and then increased. However, if considered in the context that this amino acid is essential for Lxx possibly as a result of the mutational loss of function of the cysteine synthase (*cysK*) gene [11], the reduction at 30 DAI, which was not observed in the S genotype, would significantly impact on its growth potentially indicating an important mechanism to reduce the population levels of Lxx in resistant genotypes.

### 3.5. Bacterial Inoculation Decreases the Abundance of Abscisic in the Xylem Sap of the Susceptible Variety

Inoculation with the pathogen induced few metabolic changes in the sap of both varieties. Noteworthy is the significant decrease at 30 DAI in the abundance of abscisic acid (ABA) of the susceptible variety, but this finding must be approached with caution due to the relatively large standard deviation values and because ABA levels have been reported to increase in inoculated plants of susceptible varieties [51], although at later times (90, 120, 150 and 180 DAI) than the one reported here. Notwithstanding, its potential involvement as a mediator of defense responses to Lxx deserves further investigation as this phytohormone acts on a pathogen-specific manner either as a suppressor or an enhancer of the immune responses [52]. Thus, considering the latter scenario, the reduction of ABA levels by a yet unknown mechanism in S would be beneficial to *L. xyli* subsp. *xyli*.

### 3.6. Transcriptomic Data Expand the Connection between Metabolic Changes of the Resistant Genotype Related to Pathogen Inoculation

Omics analyses provide complementary information of the mechanisms operating at different levels to control biological processes and the integration of their data strongly supports translational research. Our previous transcriptomic study of Lxx-inoculated plants of the same R variety used in this study (SP80-3290) analyzed at 30 and 60 DAI [14] allowed to highlight some examples of the complex correspondences between gene transcription and metabolite intensity. For instance, the differential abundances of the eight amino acids did not coincide with altered expressions of genes directly implicated in their synthesis. However, at both times, genes related to the synthesis of methionine and of the defense signaling hormone ethylene were all upregulated upon inoculation, which is interesting given that threonine, homoserine, and cysteine are precursors of methionine. Thus, by considering both sets of data, it is possible to broaden the connection between the synthesis of amino acids and the production of ethylene in response to inoculation, indicating the need for more detailed investigations on the processes underlying these changes as they appear to play a central role in the response of a resistant genotype to *L. xyli.* subsp. *xyli*.

Other correspondences between metabolomics and transcriptomics were found at the common time of 30 DAI in the malate, caffeic acid, and synapaldehyde pathways. Malate accumulation in the leaves at 30 DAI correlated with the increased expression levels of a gene coding for the enzyme (pyruvate phosphate dikinase) involved in the regeneration of malate through the conversion of pyruvate into phosphoenolpyruvate, one of the precursors of malate in the mesophyll cells. Finally, the down-expression of a gene coding for 4-coumarate CoA ligase, a key enzyme of the phenylpropanoid pathway, correlates with the lower abundances of both caffeic acid in the leaves and of sinapaldehyde in the xylem sap since the enzyme catalyzes reactions downstream (caffeic acid → caffeoyl CoA) or upstream (sinapic acid → sinapoyl CoA → sinapaldehyde) of the synthesis of these compounds.

No correspondences could be traced between the shifts in the abundances of maltose, inositol, and arabitol and the differential expression of genes involved in the metabolism of these sugars, which most likely results from data insufficiency. However, the above-discussed correspondences indicate the promising prospects of integrative omics to broaden our understanding of this challenging disease of sugarcane as more studies become available.

## 4. Materials and Methods

### 4.1. Chemicals

All unlabeled standards and pyridine were ordered from Millipore-Sigma (St. Louis, MO, USA). Labeled standards ([U-^13^C_2_]-glycine, [U-^13^C_6_]-glucose, [U-^13^C_4_]-fumarate, and ^13^C-benzoic acid) were ordered from Cambridge Isotope Laboratories, Inc (Tewksbury, MA, USA). LC-MS grade solvents (chloroform, methanol, acetonitrile) and N-Methyl-N-(trimethylsilyl) trifluoroacetamide with 1% trimethylchlorosilane (MSTFA + 1% TMCS) were obtained from Thermo Fisher Scientific (Waltham, MA, USA). All chemicals used were analytical grade unless stated otherwise.

### 4.2. Bacterial Cultivation

The CTCB07 strain of Lxx was used. A 1-mL volume of a bacterial suspension obtained from a 25% glycerol inoculum was cultivated in 250-mL flasks containing 50 mL of modified sugarcane New (MSC-New) medium [26,53]. The culture was incubated at 28 °C and 400 rpm agitation until it reached an optical density (O.D) of 0.7 (approximately 7 days; λ = 600 nm). To prepare cell suspensions for inoculations, the cultured medium was centrifugated at 3000 rpm for 20 min at room temperature to obtain a bacterial pellet, which was resuspended in 0.85% saline solution to O.D = 0.7.

### 4.3. Plant Inoculation and Bacterial Quantification

One-bud stem cuttings (setts) of the susceptible CB49-260 (S) and of the resistant SP80-3280 (R) varieties were obtained from greenhouse-grown plants infected with low Lxx titers. The setts were planted in commercial substrate (Basaplant, BASE) in a growth chamber at 25 °C and photoperiod of 12 h until the sprouted plantlets reached 1.5 cm in height (approximately six days). Plantlets were inoculated by placing 50 µL of the cell suspension on top of the surface of the sprouted buds that were cut just above the meristem with a sterile blade. Control plants were mock-inoculated with the same volume of 0.85% NaCl. After inoculation, the plantlets were transplanted to 22 L plastic pots, each pot containing 20 L of substrate (Basaplant, BASE) supplemented with 25 g of NPK (8-28-16). The inoculum was serially diluted and plated on solid MSC-New medium for the determination of its concentration.

The experiment was conducted in a greenhouse and consisted of eight treatments: two varieties inoculated or not with Lxx at two sampling times. Each treatment had four biological replicates, consisting of one pot containing a single plant; samples from leaves and from the xylem were non-destructively collected at two time points (30 and 120 DAI), comprising 32 samples of each source. Bacterial titers were quantified in the stem fluid 180 DAI by qPCR as in Carvalho et al. [15]. The fluid was collected by pressing pieces of the stem with a Lineman’s plier; 500 µL aliquots were used for DNA extraction.

### 4.4. Sample Collection

The first leaves with a visible dewlap (+1 leaves) were collected at 30 and 120 DAI, flash-frozen and kept at −80 °C until they were ground in liquid nitrogen using a pestle and a mortar and lyophilized for 48 h in Eppendorf tubes. The samples were kept at −20 °C until processing. Xylem sap samples were pipetted from the hydathodes in the form of guttation droplets every 30 min for 3 h starting at 6 AM and stored in sterile tubes kept on ice during the collection. The samples were flash-frozen and stored at −80 °C until they were aliquoted in 200 µL sub-samples into 1.5 mL tubes, lyophilized for 48 h, and preserved under the same conditions as the leaf samples.

### 4.5. Metabolomic Analyses

#### 4.5.1. Untargeted Metabolomics

Metabolomic analyses were performed at the Center for Applied Plant Sciences Targeted Metabolomics Laboratory at The Ohio State University. Untargeted metabolic profiling was performed only in leaf and xylem sap samples collected at 120 DAI to identify the main classes of metabolites present in these tissues. All treatments had four replicates, except for sap S and R of the mock-inoculated treatment, which had three replicates each.

Metabolites were extracted from the leaves with −20 °C water/methanol/chloroform solvent (1:2.5:1; *v*/*v*/*v*). Approximately 5 mg of lyophilized tissue were weighed, and the exact weight was recorded for data normalization. One mL of cold extraction solvent (chloroform/methanol/water 1:2.5:1; *v*/*v*/*v*) was added to each sample as well as 10 µL of 20 mM [U-^13^C_2_]-glycine as an internal standard. The samples were vortexed at 4 °C for 5 min and centrifuged at 17,000× *g* for 2 min. The upper colorless (polar) phase of the supernatant was collected transferred to a new tube and then methanol was evaporated with a speed-vacuum and the remaining liquid was lyophilized. Dry samples were resuspended in 250 µL methanol/water (50:50; *v*/*v*), and cleaned through a 3 kDa Amicon filtering device at 14,000× *g* for 45 min at 4 °C.

Lyophilized sap samples were resuspended in 250 µL of extraction solvent (methanol/water 2:8; *v*/*v*) and 3 µL of 2 mM [U-^13^C_2_]-glycine standard solution was added as an internal standard. The samples were vortexed at 4 °C for 5 min and centrifuged at 17,000× *g* for 2 min. The supernatant was collected and cleaned through a 0.2 µm Nanosep filtering device at 17,000× *g* for 10 min.

For both leaf and sap samples, the methanol from the filtered extracts was evaporated in a speed-vacuum, after which they were lyophilized and resuspended in methylene chloride. The extracts were dried under a flow of nitrogen gas, and then derivatized with 20 mg/mL methoxyamine hydrochloride in pyridine and MSTFA + 1% TMCS. The derivatized samples were subjected to gas chromatography-mass spectrometry (GC-MS) analysis as previously described [54], using a Thermo Trace 1310 gas chromatograph coupled to an ISQ single quadrupole mass spectrometer. The samples were separated using a TG-5MS capillary (30 m × 0.25 mm × 0.50 µm) column from Thermo Scientific. To avoid carry-over in the xylem sap sample runs, a blank (100% acetonitrile) was injected between each sample. The Xcalibur software v. 4.0 (Thermo Scientific) was used for data acquisition and processing of the GC-MS data; XCMS Online [55] was used for chromatogram alignment and peak integration. The main classes of compounds were identified using the National Institute of Standards and Technology (NIST 14) mass spectral library. Relative abundances were calculated by normalizing the area of each individual detected metabolite by ^13^C-internal standard and the weight of the leaf sample or the volume of sap extract.

#### 4.5.2. Targeted Metabolomics

For the targeted analysis of leaf extracts, amino acids, sugars, sugar alcohols, organic acids, phosphorylated metabolites, phenolic compounds, flavonoids, and hormones were quantified by liquid chromatography tandem mass spectrometry (LC-MS/MS) using a UHPLC 1290 (Agilent) for LC separation and QTRAP 5500 linear Ion Trap Quadrupole LC-MS/MS Mass Spectrometer (AB Sciex Instruments) for tandem MS. Four replicates were analyzed for all treatments, except for leaves of the mock-inoculated R variety collected at 120 DAI, which had *n* = 3.

Amino acids, sugars and sugar alcohols, organic acids, and phosphorylated compounds were extracted with boiling water as in Cocuron et al. [56]. Ten µL of a mixture containing 10 mM [U-^13^C_6_]-glucose, 1 mM [U-^13^C_4_]-fumarate, and 5 mM [U-^13^C_2_]-glycine was added to each sample as internal standard. For amino acid analyses, extracts were injected onto a Hypercarb column (100 × 2.1 mm, 5 µ pore; Thermo Fisher Scientific). Sugars and sugar alcohols were resolved using a Shodex Asahipak NH2P-50 2D column (2.0 × 150 mm) with a NH2P-50G 2A guard column (Showa Denko America). Organic acids and phosphorylated compounds were separated by a IonPac AS11 column (250 × 2 mm) with a guard column AG11 (50 × 2 mm; Dionex).

Phenolics were extracted using methanol:water (40:60 *v*/*v*), as described previously [57]. Briefly, 10 mM of ^13^C-benzoic acid was added as an internal standard to the samples. Phenolic compounds, flavonoids, and hormones were separated using a reverse phase C18 Symmetry column (4.6 × 75 mm; 3.5 µ) associated with a Symmetry C18 pre-column (3.9 × 20 mm; 5 µ; Waters).

It is important to note that no extraction was performed for the analysis of xylem sap metabolites. Sap samples were lyophilized, resuspended in 250 µL water:methanol (1:1 *v*/*v*), and the different classes of compounds were analyzed by LC-MS/MS and processed as the leaf extracts.

Data acquisition and processing were performed with Analyst v. 1.6.2 software. Metabolite quantification was performed by correlating the resulting peak area of each metabolite with its corresponding standard as previously described by Cocuron et al. [58]. First, the total amount of a sample in each run was estimated according to its dilution. Then, each metabolite peak area was corrected to the ^13^C-internal standard. Finally, each area was correlated to the concentration and integrated area of the corresponding external standard.

### 4.6. Statistical Analysis

Comparisons of mean bacterial titers (cells/mL) among treatments were performed by one-way ANOVA and Tukey’s test (*p* < 0.05) with MetaboAnalyst version 3.0 [59] available online (http://www.metaboanalyst.ca, accessed on 1 December 2017).

Comparisons of metabolite abundances were performed with MetaboAnalyst version 3.0 (Xia et al., 2009) as well. Data were normalized using Log transformation and then mean-centered. Principal component analysis (PCA), supervised partial least-squares discriminant analysis (PLS-DA), and heatmaps were used to visualize differences among treatments and their biological replicates. Three treatment contrasts designed to address different questions were defined *a priori* to compare metabolite abundances using the *t*-test (*p* < 0.05). The first one compared the metabolite concentrations of the susceptible and the resistant mock-inoculated plants (S × R) at both times to identify constitutive metabolites that could inhibit or enhance the growth of Lxx, while the remaining two compared inoculated and mock-inoculated plants within varieties (S × SI and R × RI) at both times to identify inoculation-induced compounds possibly related to resistance to Lxx.

## 5. Conclusions and Perspectives

Lxx inoculation and bacterial quantifications confirmed the susceptibility of CB49-260 and the resistance of SP80-3280 to bacterial growth. Untargeted and targeted metabolomic profiling of leaf and xylem sap of mock-inoculated plants revealed differential abundances of metabolites between varieties mainly in the leaves; the susceptible presented higher abundances of amino acids and the resistant of phenolics, flavonoids, and salicylic acid. Considering the effects of these metabolites on the physiology of bacteria, we propose that their abundances regulate the populational levels of *L. xyli* subsp. *xyli* in sugarcane. In addition, some alterations in response to inoculation also act as contributing factors, such as the shifts in the abundances of phenolics and amino acids, and of the signaling molecules sorbitol and glycerol.

Further experiments are needed to validate the secondary metabolites here discussed as markers of resistance to RS and integrating omics and physiological data will provide more background knowledge to define candidate metabolites. Associative studies should be performed relating natural variations in the concentrations of these compounds with variable degrees of resistance to *L. xyli* subsp. *xyli*. using a well-characterized panel of sugarcane genotypes. Additionally, in vitro experiments can further demonstrate their effects on bacterium growth and viability. The knowledge resulting from these studies will shed more light on this still poorly understood pathosystem. Additionally, because sugarcane breeding is extremely challenging at the genic level due to the complex genetic nature of its polyploid genome [60], it is important to determine resistance-related molecules and establishing biomarkers for metabolite-based selection will improve the breeding process for resistant genotypes to ratoon stunt, a novel and necessary approach to reduce crop losses caused by this global disease.

## Data Availability

The data presented in this study are available in article.

## Supplementary Materials

The following are available online at https://www.mdpi.com/article/10.3390/metabo11040234/s1, Table S1: Mass/charge ratio (*m/z*), retention time (RT), and average relative intensity ± standard deviation of features detected by untargeted metabolomics in leaves of a susceptible (S) or resistant (R) sugarcane variety mock-inoculated or inoculated with Lxx (I) at 120 DAI from leaves extracts or sap (*n* = 4, except for sap S and R mock-inoculated, which had *n* = 3), Table S2: Metabolites of sugarcane sap mock-inoculated or Lxx-inoculated (I) from susceptible (S) or resistant (R) plants to RS at 30 and 120 days after inoculations (DAI), Table S3: Metabolites of sugarcane leaves mock-inoculated susceptible (S) or resistant (R) to RSD at 30 and 120 days after inoculations (DAI), Table S4: Summary of metabolites significant (*p* < 0.05) in at least one comparison of sugarcane leaves mock-inoculated or Lxx-inoculated (I) susceptible (S) or resistant (R) to RSD at 30 and 120 days after inoculations (DAI), Table S5: Metabolites of sugarcane leaves susceptible (S) to RSD mock-inoculated or Lxx-inoculated (I) at 30 and 120 days after inoculations (DAI), Table S6: Metabolites of sugarcane leaves resistant (R) to RSD mock-inoculated or Lxx-inoculated (I) at 30 and 120 days after inoculations (DAI). Abbreviations for supplemental Tables S3–S5: GABA, gamma aminobutyric acid; UMP, uridine monophosphate; ADP, adenosine diphosphate; CTP, cytidine triphosphate; UDP-glucose, uracil diphosphate glucose; CMP, cytidine monophosphate; AMP, adenosine monophosphate; IMP, inosine monophosphate; GMP, guanosine monophosphate; UDP, uridine diphosphate; GDP, guanosine diphosphate; UTP, uridine triphosphate; ATP, adenosine triphosphate; GTP, guanosine triphosphate; CDP, cytidine diphosphate; UDP-N-Galactosamine, uridine diphosphate-N-galactosamine; 3-CQA, 3-caffeoylquinic acid; 4-CQA, 4-caffeoylquinic acid; 5-CQA, 5-caffeoylquinic acid; FQA 1–4, isomers 1, 2, 3 and 4 of feruloylquinic acid; pCoQA 1 and 2, isomers 1 and 2 of p-coumaroylquinic acid.

## References

[1] Li W.-F., Shen K., Huang Y.-K., Wang X.-Y., Yin J., Luo Z.-M., Zhang R.-Y., Shan H.-L. (2014). Incidence of sugarcane ratoon stunting disease in the major cane-growing regions of China. Crop Prot..

[2] Davis M.J., Gillaspie A.G., Harris R.W., Lawson R.H. (1980). Ratoon stunting disease of sugarcane: Isolation of the causal bacterium. Science.

[3] Teakle D., Smith P., Steindl D. (1973). Association of a small coryneform bacterium with the ratoon stunting disease of sugar-cane. Aust. J. Agric. Res..

[4] Croft B.J. (2002). A method for rating sugarcane cultivars for resistance to ratoon stunting disease based on an enzyme-linked immunoassay. Australas. Plant Pathol..

[5] Fegan M., Croft B.J., Teakle D.S., Hayward A.C., Smith G.R. (1998). Sensitive and specific detection of *Clavibacter xyli* subsp. *xyli*, causal agent of ratoon stunting disease of sugarcane, with a polymerase chain reaction-based assay. Plant Pathol..

[6] Urashima A.S., Silva M.F., Correia J.J., Moraes M.C., Sibgh A.V., Sainz M.B. (2017). Prevalence and severity of ratoon stunt in commercial Brazilian sugarcane fields. Plant Dis..

[7] Fu H.Y., Sun S.R., Wang J.D., Ahmad K., Wang H.B., Chen R.K., Gao S.J. (2016). Rapid and quantitative detection of *Leifsonia xyli* subsp. *xyli* in sugarcane stalk juice using a real-time fluorescent (TaqMan) PCR assay. Biomed Res. Int..

[8] Urashima A.S., Marchetti L.B.L. (2013). Incidence and Severity of *Leifsonia xyli* subsp. *xyli* Infection of Sugarcane in Sao Paulo State, Brazil. J. Phytopathol..

[9] Young A.J. (2016). Seedbed inspections underestimate the overall incidence of ratoon stunting disease. Int. Sugar J..

[10] Grisham M. (1991). Effect of ratoon stunting disease on yield of sugarcane grown in multiple three-year plantings. Phytopathology.

[11] Monteiro-Vitorello C.B., Zerillo M.M., Van Sluys M.-A., Camargo L.E.A., Jackson R. (2009). Genome sequence-based insights into the biology of the sugarcane pathogen *Leifsonia xyli* subsp. *xyli*. Plant Pathogenic Bacteria—Genomics and Molecular Biology.

[12] Quecine M.C., Silva T.M., Carvalho G., Saito S., Mondin M., Teixeira-Silva N.S., Camargo L.E.A., Monteiro-Vitorello C.B. (2016). A stable *Leifsonia xyli* subsp. *xyli* GFP-tagged strain reveals a new colonization niche in sugarcane tissues. Plant Pathol..

[13] Young A.J. (2016). Possible origin of ratoon stunting disease following interspecific hybridization of *Saccharum* species. Plant Pathol..

[14] Cia M.C., de Carvalho G., Azevedo R.A., Monteiro-Vitorello C.B., Souza G.M., Nishiyama-Junior M.Y., Lembke C.G., Antunes de Faria R.S.d.C., Marques J.P.R., Melotto M. (2018). Novel insights into the early stages of ratoon stunting disease of sugarcane inferred from transcript and protein analysis. Phytopathology.

[15] Carvalho G., da Silva T.G.E.R., Munhoz A.T., Monteiro-Vitorello C.B., Azevedo R.A., Melotto M., Camargo L.E.A. (2016). Development of a qPCR for *Leifsonia xyli* subsp. *xyli* and quantification of the effects of heat treatment of sugarcane cuttings on Lxx. Crop Prot..

[16] Urashima A.S., Grachet N.G. (2012). Métodos de detecção de *Leifsonia xyli* subsp. *xyli* e efeito da termoterapia na brotação das gemas de diferentes variedades de cana-de-açúcar. Trop. Plant Pathol..

[17] Hoy J.W., Grisham M.P., Damann K.E. (1999). Spread and increase of ratoon stunting disease of sugarcane and comparison of disease detection methods. Plant Dis..

[18] Harrison N.A., Davis M. (1988). Colonization of vascular tissues by *Clavibacter xyli* subsp. *xyli* in stalks of sugarcane differing in susceptibility to ratoon stunting disease. Phytopathology.

[19] Gillaspie A.G., Flax G., Koike H. (1976). Relationship between numbers of diagnostic bacteria and injury by ratoon stunting disease in sugarcane. Plant Dis. Rep..

[20] McFarlane S. (2002). The relationship between extent of colonisation by *Leifsonia xyli* subsp. *xyli* and yield loss in different sugarcane varieties. Proc. S. Afr. Sugar Technol. Assess.

[21] Dal-Bianco M., Carneiro M.S., Hotta C.T., Chapola R.G., Hoffmann H.P., Garcia A.A.F., Souza G.M. (2012). Sugarcane improvement: How far can we go?. Curr. Opin. Biotechnol..

[22] Bennett R.N., Wallsgrove R.M. (1994). Secondary metabolites in plant defence mechanisms. New Phytol..

[23] Lattanzio V., Lattanzio V.M.T., Cardinali A., Amendola V. (2006). Role of Phenolics in the Resistance Mechanisms of Plants against Fungal Pathogens and Insects.

[24] Dixon R.A. (2001). Natural products and plant disease resistance. Nature.

[25] Haapalainen M., Mattinen J., Metzler M. (2000). The growth of a plant-parasitic bacterium, *Clavibacter xyli* subsp. *cynodontis*, is enhanced by xylem fluid components. Physiol. Mol. Plant Pathol..

[26] Monteiro-Vitorello C.B., Camargo L.E.A., Van Sluys M.A., Kitajima J.P., Truffi D., Do Amaral A.M., Harakava R., De Oliveira J.C.F., Wood D., De Oliveira M.C. (2004). The genome sequence of the gram-positive sugarcane pathogen *Leifsonia xyli* subsp. *xyli*. Mol. Plant-Microbe Interact..

[27] Davis M.J., Dean J.L., Harrison N.A. (1988). Quantitative variability of *Clavibacter xyli* subsp. *xyli* populations in sugarcane cultivars differing in resistance to ratoon stunting disease. Phytopathology.

[28] Rossler L. The effects of ratoon stunting disease on three sugarcane varieties under different irrigation regimes. Proceedings of the International Society of Sugarcane Technologists.

[29] Ngaruiya P.N., Shipton W.A., Coventry R. Ratoon stunting disease of sugarcane as influenced by environmental stressors. Proceedings of the 2005 Conference of the Australian Society of Sugar Cane Technologists.

[30] Alvarez S., Marsh E.L., Schroeder S.G., Schachtman D.P. (2008). Metabolomic and proteomic changes in the xylem sap of maize under drought. Plant. Cell Environ..

[31] Coutinho I.D., Baker J.M., Ward J.L., Beale M.H., Creste S., Cavalheiro A.J. (2016). Metabolite profiling of sugarcane genotypes and identification of flavonoid glycosides and phenolic acids. J. Agric. Food Chem..

[32] Schurr U. (1998). Xylem sap sampling—new approaches to an old topic. Trends Plant Sci..

[33] Rellán-Álvarez R. (2011). Metabolite profile changes in xylem sap and leaf extracts of strategy I plants in response to iron deficiency and resupply. Front. Plant Sci..

[34] Radkov A.D., Moe L.A. (2014). Bacterial synthesis of d-amino acids. Appl. Microbiol. Biotechnol..

[35] Whitehead N.A., Barnard A.M.L., Slater H., Simpson N.J.L., Salmond G.P.C. (2001). Quorum-sensing in Gram-negative bacteria. FEMS Microbiol. Rev..

[36] Vollmer W., Blanot D., De Pedro M.A. (2008). Peptidoglycan structure and architecture. FEMS Microbiol. Rev..

[37] Kim D., Woo H.M. (2018). Deciphering bacterial xylose metabolism and metabolic engineering of industrial microorganisms for use as efficient microbial cell factories. Appl. Microbiol. Biotechnol..

[38] Faria R.S.C.A., Cia M.C., Monteiro-Vitorello C.B., Azevedo R.A., Camargo L.E.A. (2020). Characterization of genes responsive to osmotic and oxidative stresses of the sugarcane bacterial pathogen *Leifsonia xyli* subsp. *xyli*. Brazilian J. Microbiol..

[39] Lou Z., Wang H., Zhu S., Ma C., Wang Z. (2011). Antibacterial activity and mechanism of action of chlorogenic acid. J. Food Sci..

[40] Ngadze E., Icishahayo D., Coutinho T.A., Vand der Waals J.E. (2012). Chlorogenic acid, and total soluble phenols in resistance of potatoes to soft rot. Plant Dis..

[41] Niggeweg R., Michael A.J., Martin C. (2004). Engineering plants with increased levels of the antioxidant chlorogenic acid. Nat. Biotechnol..

[42] Mierziak J., Kostyn K., Kulma A. (2014). Flavonoids as important molecules of plant interactions with the environment. Molecules.

[43] Grant M.R., Jones J.D.G. (2009). Hormone (dis)harmony moulds plant health and disease. Science.

[44] Yang Y., Qi M., Mei C. (2004). Endogenous salicylic acid protects rice plants from oxidative damage caused by aging as well as biotic and abiotic stress. Plant J..

[45] Meng D., Li C., Park H.J., González J., Wang J., Dandekar A.M., Turgeon B.G., Cheng L. (2018). Sorbitol modulates resistance to Alternaria alternata by regulating the expression of an NLR resistance gene in apple. Plant Cell.

[46] Qian Y., Tan D.-X., Reiter R.J., Shi H. (2015). Comparative metabolomic analysis highlights the involvement of sugars and glycerol in melatonin-mediated innate immunity against bacterial pathogen in Arabidopsis. Sci. Rep..

[47] Halder T., Upadhyaya G., Roy S., Biswas R., Das A., Bagchi A., Agarwal T., Ray S. (2019). Glycine rich proline rich protein from Sorghum bicolor serves as an antimicrobial protein implicated in plant defense response. Plant Mol. Biol..

[48] Zhang Y., Smith P., Maximova S.N., Guiltinan M.J. (2015). Application of glycerol as a foliar spray activates the defence response and enhances disease resistance of Theobroma cacao. Mol. Plant Pathol..

[49] Zhang X.-Q., Liang Y.-J., Zhu K., Wu C.-X., Yang L.-T., Li Y.-R. (2017). Influence of inoculation of *Leifsonia xyli* subsp. *xyli* on photosynthetic parameters and activities of defense enzymes in sugarcane. Sugar Tech.

[50] Rojas C.M., Senthil-Kumar M., Tzin V., Mysore K.S. (2014). Regulation of primary plant metabolism during plant-pathogen interactions and its contribution to plant defense. Front. Plant Sci..

[51] Zhang X., Chen M., Liang Y., Xing Y., Yang L., Chen M., Comstock J.C., Li Y., Yang L. (2016). Morphological and physiological responses of sugarcane to *Leifsonia xyli* subsp. *xyli* infection. Plant Dis..

[52] Bari R., Jones J.D.G. (2009). Role of plant hormones in plant defence responses. Plant Mol. Biol..

[53] Davis M.J., Gillaspie JR A.G., Vidaver A.K., Harris R.W. (1984). Clavibacter: A new genus containing some phytopathogenic coryneform bacteria, including *Clavibacter xyli* subsp. *xyli* sp. nov., subsp. nov. and *Clavibacter xyli* subsp. *cynodontis* subsp. nov., pathogens that cause ratoon stun. Int. J. Syst. Bacteriol..

[54] Tsogtbaatar E., Cocuron J.-C., Sonera M.C., Alonso A.P. (2015). Metabolite fingerprinting of pennycress (*Thlaspi arvense* L.) embryos to assess active pathways during oil synthesis. J. Exp. Bot..

[55] Tautenhahn R., Patti G.J., Rinehart D., Siuzdak G. (2012). XCMS Online: A web-based platform to process untargeted metabolomic data. Anal. Chem..

[56] Cocuron J.-C., Anderson B., Boyd A., Alonso A.P. (2014). Targeted metabolomics of *Physaria fendleri*, an industrial crop producing hydroxy fatty acids. Plant Cell Physiol..

[57] Cocuron J.-C., Casas M.I., Yang F., Grotewold E., Alonso A.P. (2019). Beyond the wall: High-throughput quantification of plant soluble and cell-wall bound phenolics by liquid chromatography tandem mass spectrometry. J. Chromatogr. A.

[58] Cocuron J.-C., Alonso A.P. (2014). Liquid chromatography tandem mass spectrometry for measuring 13C-labeling in intermediates of the glycolysis and pentose phosphate pathway. Methods Mol. Biol..

[59] Xia J., Psychogios N., Young N., Wishart D.S. (2009). MetaboAnalyst: A web server for metabolomic data analysis and interpretation. Nucleic Acids Res..

[60] Raboin L.-M., Oliveira K.M., Lecunff L., Telismart H., Roques D., Butterfield M., Hoarau J.-Y., D‘Hont A. (2006). Genetic mapping in sugarcane, a high polyploid, using bi-parental progeny: Identification of a gene controlling stalk colour and a new rust resistance gene. Theor. Appl. Genet..

